# Primary Leiomyosarcoma of the Gallbladder

**DOI:** 10.1155/2020/3603689

**Published:** 2020-06-22

**Authors:** Anwar Chammout, Mike Ghabally, Christina Masri, Nour Tantak, Fadi Ward, Ghefar Omar

**Affiliations:** ^1^Department of Oncology, University of Aleppo, Faculty of Medicine, Aleppo, Syria; ^2^University of Aleppo, Faculty of Medicine, Aleppo, Syria; ^3^Department of Pathology, University of Aleppo, Faculty of Medicine, Aleppo, Syria

## Abstract

Leiomyosarcoma (LMS) of the gallbladder is an extremely rare entity. Most reported cases were mistakenly diagnosed preoperatively as cholecystitis with or without cholelithiasis. We believe that our article demonstrates the 5^th^ case of gallbladder LMS that was suspected preoperatively to be malignant rather than cholecystitis, which fortunately led to radical resection of the tumor instead of simple cholecystectomy. However, the definitive diagnosis relies exclusively on histological and immunohistochemical techniques. We present a case of a 62-year-old Caucasian female complaining of signs and symptoms suggestive for cholecystitis. On ultrasonography, the gallbladder appeared enlarged and filled with a necrotic mass; thus, the presence of adenocarcinoma was suspected. Multislice Computerized Tomography (MSCT) demonstrated no distant metastasis. An extensive radical cholecystectomy was performed, and histological techniques confirmed a leiomyosarcoma diagnosis. In addition, adjuvant chemotherapy of doxorubicin and ifosfamide was administrated. The patient was on follow-up for 2 years and is doing well till date. The discrimination of gallbladder sarcoma preoperatively remains a clinical and radiological challenge. Although radical resection of the tumor remains the mainstay of the treatment, we believe that adjuvant chemotherapy should be administrated in such cases. However, further studies are required in this field.

## 1. Introduction

Primary sarcoma of the gallbladder is an extremely rare diagnosis, and one of its common subtypes is leiomyosarcoma (LMS).

This neoplasm was first reported in 1879, while the first detailed description was made by Landsteiner in 1904 [1]. Most reported cases of LMS are made with a primitive diagnosis of cholecystitis with or without cholelithiasis in female patients in their sixth decade [2, 3].

Our search through the medical literature revealed only 22 other case of LMS. Therefore, we believe that our case is the 23^rd^ in this series. This neoplasm is rarely diagnosed preoperatively. The accurate diagnosis is only established with histological and immunohistochemical techniques after cholecystectomy [2, 4].

## 2. Case Presentation

A 62-year-old Caucasian woman was admitted to the hospital complaining of abdominal pain in the right upper quadrant that started a month ago. The pain was severe, nonradiating, and colic. It was exacerbated by eating fatty meals and was responded to heavy analgesics. She also suffered from nausea, bilious vomiting, constipation, fever reaching 39°C that lasted for 10 days, chills, anorexia, and fatigue. There was no history of weight loss, pallor, jaundice, smoking, or alcohol consumption. Formerly, she was in good health as she did not report any previous illnesses or surgeries and her family history was unremarkable.

On examination, Murphy's sign was positive and there was tenderness over the gallbladder area with a firm palpable mass.

**Figure 1:**
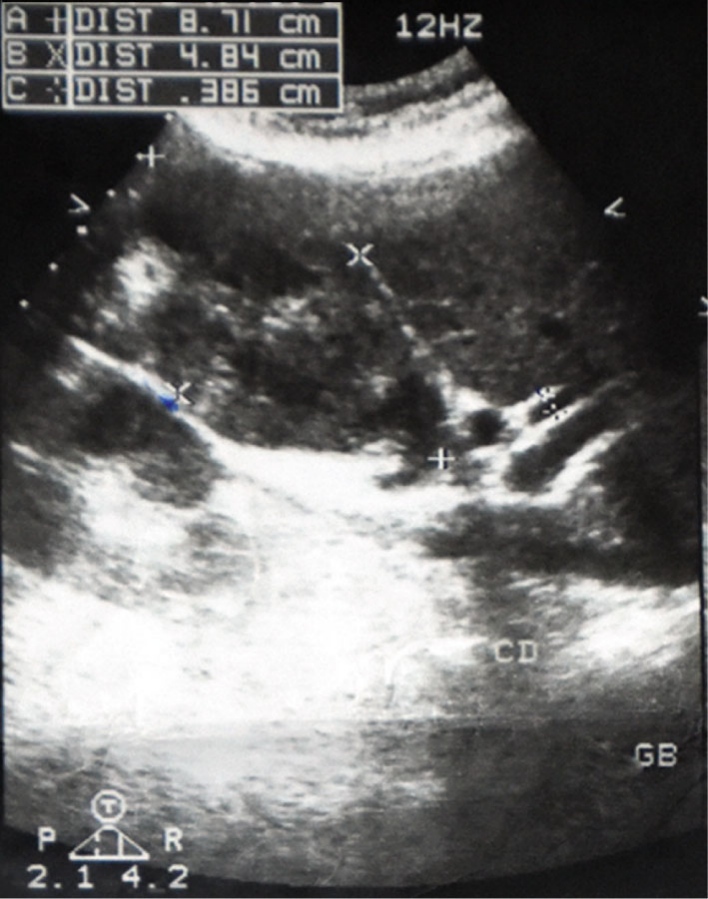
Transabdominal ultrasonography.

Laboratory workup showed a normal hemogram and liver function tests. On abdominal ultrasonography, the gallbladder measured 8.7 × 4.8 cm and was filled with necrotized mass and all edges of the gallbladder were clear except for the bottom (Figure 1). Therefore, the presence of a gallbladder adenocarcinoma was suspected. Spleen, pancreas, kidney, and liver were normal. MSCT demonstrated the same findings, and there was no metastasis.

As a result, a provisional diagnosis of gallbladder adenocarcinoma was made and extensive cholecystectomy that involved cholecystectomy, main bile duct resection, and regional lymph node dissection was performed. Intraoperative findings showed no liver metastasis, ascites, peritoneal, or omental deposits. Porta was free, and there was no colonic or duodenal infiltration and no enlarged pericholedochal, paraaortic, or aortocaval lymph nodes.

**Figure 2:**
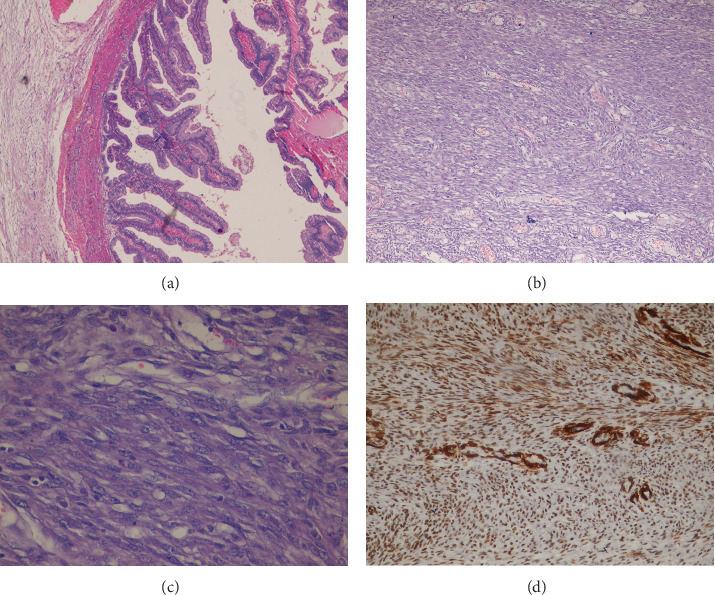
Pathohistochemical profile. (a) Ligand: normal gallbladder mucosa. (b, c) H&E stain shows proliferation of spindle cells arranged in interlacing bundles, pronounced nuclear atypia, pleomorphism, and many atypical mitotic figures (×200 and ×400, respectively). (d) SMA stain: the neoplastic cells show a strong cytoplasmic staining reaction (×200).

On microscopy, the tumor consisted of diffuse proliferation of pleomorphic spindle to oval tumor cells with marked variation in size and shape and the presence of many abnormal mitoses and large hyperchromatic nuclei (Figure 2). Furthermore, the gallbladder epithelium mucosa was congested and ulcerated with a mild degree of lymphocytic infiltration.

Immunohistochemical investigations were strongly positive for smooth muscle actin (SMA) and desmin and negative for CK, CD117, and CD34 (Figure 2). Therefore, we put the conclusive diagnosis as primary leiomyosarcoma of the gallbladder. However, we could not perform any further immunohistochemical markers.

Although there is no sufficient evidence regarding the effectiveness of adjuvant chemotherapy with or without radiotherapy, our patient received 6 cycles of adjuvant chemotherapy as the following regimen: (doxorubicin (day 1: 60 mg/m^2^)+ifosfamide (days 1-3: 2000 *μ*g/m^2^); repeated monthly for 6 cycles). No serious adverse effect or toxicity has occurred. However, radiotherapy is not available in our city. The patient was on follow-up for 2 years; there was no clinical or radiological evidence of recurrence or relapse as observational MSCT scans were clear for any local invasion or distal metastasis, and the patient is doing well till date.

## 3. Discussion

Sarcoma of the gallbladder is an extremely rare entity that was reported less than 200 times in the medical literature [5]. Older cases agreed that LMS was the most common subtype of Primary Gallbladder Sarcoma (PGBS). On the other hand, recent series revealed that LMS is not as common as previously reported with MFH (malignant fibrous histiocytoma) being the most common subtype [6, 7].

Most of the patients in the reported cases are females (ratio 5 : 1) and mainly in their 6^th^ and 7^th^ decades of life [4–6, 8].

In our search among almost 200 reported PGBSs, there were only 6 cases that were suspected to be malignant preoperatively. Only 4 had the definitive diagnosis of LMS. Thus, our case represents the 5^th^ case in this series [4, 6, 7]. However, the definitive diagnosis relies exclusively on histological and immunohistochemical techniques [8, 9].

Patients with GB sarcoma suffer from nonspecific symptoms that can be divided into two categories [7, 10]. The first category includes symptoms of gallbladder cholelithiasis, including right upper quadrant pain, nausea, vomiting, jaundice, and fever. The second category includes symptoms related to the malignant nature of the disease such as weight loss, fatigue, and anorexia [6–8, 10].

LMS coexists with cholelithiasis in 82% of cases. Therefore, it was related as a primitive factor, although the casual relationship remains circumstantial and unclear [2].

Radiological findings on CT scan and ultrasonography may include a dilated gallbladder, an irregular thickening of the wall, and a necrotizing mass protruding into the lumen [1, 5]. The larger size of the gallbladder, the presence of necrosis, and the polypoidal shape of the tumor should increase the preoperative suspicion of malignancy [6]. It is noteworthy that the diagnosis of such cases and the identification of histopathological subtypes depend on the extent of expertise of the radiologist and pathologist.

The differential diagnosis of a solid polypoidal gallbladder mass is wide and includes adenomas, adenocarcinomas, and neoplastic polyps such as cholesterol polyps, inflammatory polyps, and adenomatous hyperplasia [3, 5]. The definitive diagnosis relies on both morphological features on H&E staining and immunostains [8, 9].

The extensive differential diagnosis of spindle cell tumors of the gallbladder includes leiomyosarcoma, rhabdomyosarcoma, fibrosarcoma, neurosarcoma, epithelioid angiosarcoma, gastrointestinal stromal tumors (GIST), carcinosarcoma, and primary or metastatic undifferentiated carcinoma [1, 3, 5, 7].

Immunohistochemical techniques are the best way to differentiate between sarcomas. The spindle cells in LMS are arranged in a fascicular pattern and have eosinophilic cytoplasm with round to oval nuclei [8]. As previously mentioned, the high positivity for SMA in our case favored the smooth muscle origin of the tumor [1, 10]. In addition, desmin was positive. However, immunostains were negative for CK, CD34, and CD117.

LMS of the gallbladder has a very poor prognosis with a 5-year survival rate less than 5% [2, 5, 8, 9]. This is due to the highly malignant nature, rapid progression, and early metastasis [5, 10]. Almost 75% of all patients have liver involvement at the time of diagnosis [2, 7–9]. However, our patient did not have any liver invasion or distal metastasis.

The treatment of choice remains radical cholecystectomy, and in advanced cases, regional lymph node dissection should be performed [2, 5, 8]. Adjuvant chemotherapy with or without radiotherapy has prolonged the overall survival rate in many reported cases of LMS [2, 3, 5, 9]. In our case, the patient underwent and extended radical cholecystectomy with 6 cycles of adjuvant chemotherapy (doxorubicin and ifosfamide) and was on follow-up for 2 years without any clinical or radiological evidence of relapse.

## 4. Conclusion

LMS of the gallbladder is rare and hard to be differentiated from other malignant subtypes. In most cases, it is falsely diagnosed as cholelithiasis and cholecystitis.

Although radical resection of the tumor remains the mainstay of the treatment, we believe that adjuvant chemotherapy should be administrated in such cases. However, further studies are required in this field.

## Data Availability

Data availability is not applicable.

## Abbreviations

CK: Cytokeratin
GB: Gallbladder
GIST: Gastrointestinal stromal tumors
H&E: Hematoxylin and eosin
LMS: Leiomyosarcoma
MFH: Malignant fibrous histiocytoma
MSCT: Multislice Computerized Tomography.
PGBS: Primary Gallbladder Sarcoma
SMA: Smooth muscle actin.
